# Interleukin-6 promotes primitive endoderm development in bovine blastocysts

**DOI:** 10.1186/s12861-020-00235-z

**Published:** 2021-01-12

**Authors:** Lydia K. Wooldridge, Alan D. Ealy

**Affiliations:** Department of Animal and Poultry Sciences, Virginia Polytechnic Institute and State University, 3430 Litton-Reaves Hall (0306), Blacksburg, VA 24060 USA

**Keywords:** Blastocyst, Interleukin-6, Epiblast, Primitive endoderm, Inner cell mass

## Abstract

**Background:**

Interleukin-6 (IL6) was recently identified as an embryotrophic factor in bovine embryos, where it acts primarily to mediate inner cell mass (ICM) size. This work explored whether IL6 affects epiblast (EPI) and primitive endoderm (PE) development, the two embryonic lineages generated from the ICM after its formation. Nuclear markers for EPI (NANOG) and PE (GATA6) were used to differentiate the two cell types.

**Results:**

Increases (*P* < 0.05) in total ICM cell numbers and PE cell numbers were detected in bovine blastocysts at day 8 and 9 post-fertilization after exposure to 100 ng/ml recombinant bovine IL6. Also, IL6 increased (*P* < 0.05) the number of undifferentiated ICM cells (cells containing both PE and EPI markers). The effects of IL6 on EPI cell numbers were inconsistent. Studies were also completed to explore the importance of Janus kinase 2 (JAK2)-dependent signaling in bovine PE cells. Definitive activation of STAT3, a downstream target for JAK2, was observed in PE cells. Also, pharmacological inhibition of JAK2 decreased (*P* < 0.05) PE cell numbers.

**Conclusions:**

To conclude, IL6 manipulates ICM development after EPI/PE cell fates are established. The PE cells are the target for IL6, where a JAK-dependent signal is used to regulate PE numbers.

**Supplementary Information:**

The online version contains supplementary material available at 10.1186/s12861-020-00235-z.

## Background

Interleukin-6 (IL6) is a cytokine that is best known as an innate immune response molecule [1]. However, the detection of *IL6* transcripts within bovine blastocysts, peri-implantation conceptuses and the bovine oviduct and uterus during early pregnancy suggests that it may also serve as an embryotrophic factor, or embryokine [2–4]. *IL6* is the most abundantly expressed member of the IL6 cytokine family in bovine blastocysts [3], which also includes other interleukins (e.g. IL11, IL27, IL31), cardiotrophin-1, oncostatin, and leukemia inhibitor factor (LIF). Bovine blastocysts also express the IL6-specific receptor subunit (*IL6R*) and the common IL6 family receptor subunit (*IL6ST*, also known as GP130), and *IL6R* is the most abundantly expressed ligand-specific receptor subunit within this cytokine family in bovine blastocysts [3].

Recent work from this group described that IL6 can influence in vitro-produced (IVP) bovine embryo development [3, 5]. However, IL6 acts differently from most other embryokines, where it targets the inner cell mass (ICM) and causes nearly a doubling in cell numbers [3, 5]. Problems with ICM development likely contribute to the high rate of failed pregnancies in transferred IVP bovine embryos [6]. These problems include reductions in ICM cell numbers, increases in the incidence of apoptosis within the ICM, and retarded post-transfer development of the embryonic disk, which develops from the ICM and produces all embryonic tissues and the yolk sac and allantoic membranes [7–11].

Another key finding of previous work was describing that IL6 may function through Janus kinase 2 (JAK2) to influence ICM development. Various downstream intracellular signaling pathways can be mediated by IL6 (e.g. mitogen-activated protein kinase [MAPK], phosphoinositide 3-kinase [PI3K]), but arguably the best known IL6-mediated signaling system involves JAK-induced phospho-activation of signaling transductor and activator of transcription 3 (STAT3) [12]. Exposure to IL6 causes a rapid phosphorylation, dimerization and nuclear localization of STAT3 within the ICM cells of bovine blastocysts [3]. This activity is specific for the ICM. No comparable IL6-induced STAT3 activation is observed in trophectoderm (TE) cells. Also, pharmacological inhibition of JAK2 prevents ICM development in bovine embryos [3, 13], and IL6 supplementation is not able to overcome this inhibition [3].

The work presented herein set out to expand on these recent findings by examining how IL6 influences development of the epiblast (EPI) and primitive endoderm (PE) lineages within the ICM. Soon after blastocyst formation, cells within the ICM differentiate into the EPI and PE lineages. This developmental event was initially described in the mouse [14], where ICM cells contain the ability to develop into either cell type, and their specification is dependent on each ICM cell’s sensitivity to embryo-derived fibroblast growth factor 4 (FGF4). Those that contain ample responsiveness to FGF4 due to the abundance of its receptor, FGFR2, develop into PE cells whereas those that contain little or no response to FGF4 develop into EPI cells [15, 16]. This specification occurs randomly throughout the ICM to form a scattered, “salt & pepper” distribution of differentiated cells when stained for the homeobox protein, NANOG (marker of EPI) and GATA binding protein 6 (GATA6; marker for PE) [17]. Soon after their specification, PE cells migrate to the base of the ICM to form the hypoblast layer, which will then expand underneath the TE to form the yolk sac [14]. The same basic events occur in bovine blastocysts, although it appears that embryo-derived FGF2 and FGF4 are equipotent at controlling PE specification [18, 19]. Differentiation of EPI and PE lineages begins at day 8–9 post-fertilization in IVP bovine blastocysts [18, 19].

The following work was completed to describe how IL6 supplementation affects development of the EPI and PE lineages in IVP bovine blastocysts. The work also explored whether JAK2 was involved with IL6 responses at this period of development.

## Results

### Supplementation with IL6 influences ICM cell numbers

**Fig. 1:**
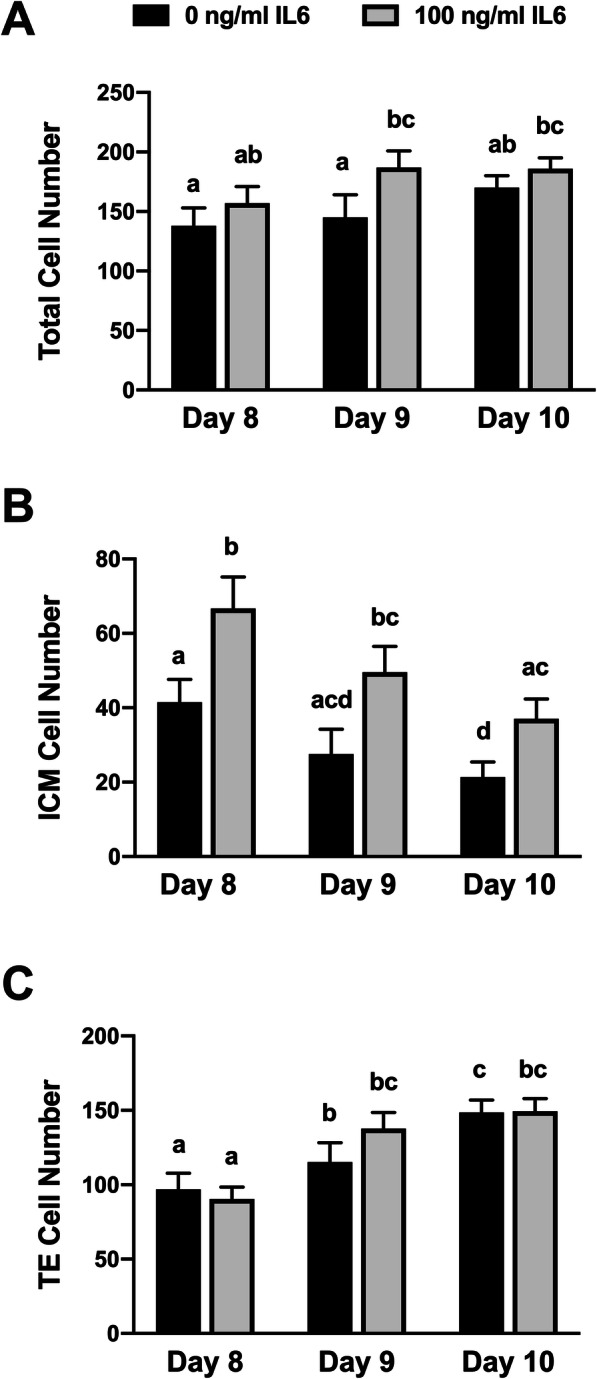
Changes in ICM and TE cell numbers after exposure to IL6. Blastocysts were cultured in SOF-BE1 until day 8, 9 or 10 post-fertilization, then differential cell staining was completed to discriminate ICM cells (CDX2^−^ / DAPI^+^) from TE cells (CDX2^+^ / DAPI^+^). **Panel a***:* Total, ICM and TE cell numbers on day 8, 9 and 10 post-fertilization (*n* = 11–18 blastocysts/group over 3 replicate studies). Different superscripts within each panel indicate differences (*P* < 0.05). **Panel b-c***:* Blastocysts were incubated with 0 or 100 ng/ml IL6 for 24 h prior to fixing and immunostaining at day 8, 9 or 10 post-fertilization (*n* = 11–18 blastocysts/group over 3 replicate studies). Main effects were detected for day of blastocyst development (*P* = 0.004) and IL6 treatment (*P* = 0.0003), but not day by treatment interaction was observed. The asterisks (*) indicate the increase (*P* < 0.05) in cell numbers within each day following IL6 treatment

The initial study was completed to describe whether IL6 supplementation increases embryo cell numbers when provided after blastocyst formation (Fig. 1). For this, IL6 supplementation (100 ng/ml) was provided in blastocysts recovered at day 7, 8 or 9 post-fertilization. After 24 h (day 8, 9, 10, respectively), blastocysts were processed to record total, ICM and TE cell numbers. Exposure to IL6 did not affect total cell numbers at any day (Fig. 1a), but an IL6-dependent increase (*P* < 0.05) in ICM cell numbers was detected at each day (Fig. 1b). No IL6-dependent changes in TE numbers were observed (Fig. 1c). Cell numbers also were affected by day of collection. An increase (*P* < 0.05) in total and TE cell numbers was detected between day 8 and 10 in both IL6-treated and untreated blastocysts. However, a decrease (*P* < 0.05) in ICM cell numbers were detected between day 8 and 10 in both IL6-treated and non-treated blastocysts. No change in IL6 response on ICM cell numbers was detected between days.

### PE cell numbers decrease over time in extended blastocyst culture

**Fig. 2:**
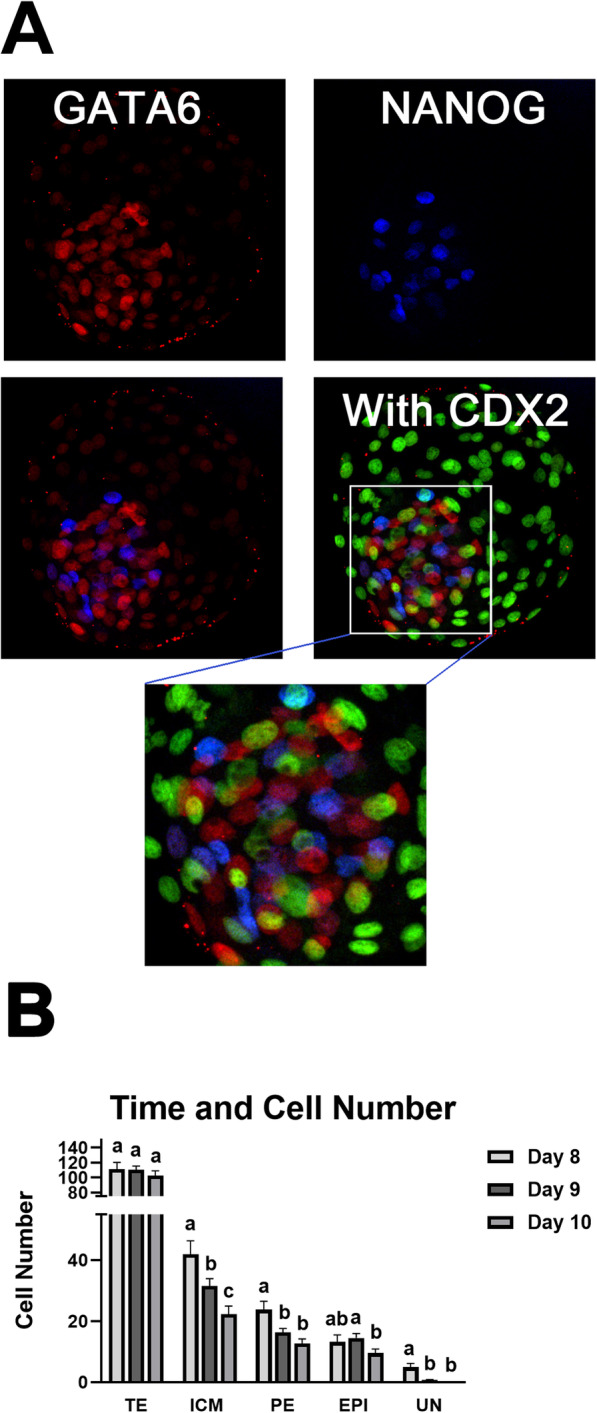
Changes in ICM cell populations over time in culture. Blastocysts were cultured in SOF-BE1 until day 8, 9 or 10 post-fertilization, then immunofluorescence staining for PE (GATA6^+^), EPI (NANOG^+^) and TE (CDX2^+^) was completed. **Panel a***:* Example of a day 8 blastocyst immunostained for GATA6 (red), NANOG (blue) and/or CDX2 (green). A higher magnification of the ICM is also shown for the triple-stained image. Cells positive for both GATA6 and NANOG were considered UN cells (purple). **Panel b***:* Analysis of TE, ICM, PE, EPI and UN cell numbers over time (*n* = 16, 51, and 40 total blastocysts from day 8, 9 and 10, respectively; 3 replicate studies). Different superscripts indicate differences for each cell type (*P* < 0.05)

A follow-up study examined how EPI and PE cell numbers were affected by day of blastocyst culture (Fig. 2). Differential cell staining was used to discriminate nuclei of cells containing markers for EPI (NANOG^+^), PE (GATA6^+^) and TE (CDX2^+^) (Fig. 2a). A subset of ICM cells contained NANOG and GATA6 co-expression. These were presumed to represent undetermined (UN) ICM cells, or cells that had not yet committed to an EPI or PE fate. While day in culture did not affect (*P* > 0.05) TE cell numbers, total ICM cell number was reduced (*P* < 0.05) with each day of extended blastocyst culture (Fig. 2b). The number of PE cells (GATA6^+^) was greater (*P* < 0.05) on day 8 than on days 9 or 10. Number of EPI cells (NANOG^+^) was not different between day 8 and 10 but showed a reduction (*P* < 0.05) in numbers between day 9 and 10. Lastly, a population of dual-positive (GATA6^+^/NANOG^+^) UN cells was detected in blastocysts at day 8 (approximately 12% of the ICM cells). The incidence of this cell population decreased (*P* < 0.05) thereafter, and very few UN cells were detected on days 9 and 10 (2.1 and 0.4% of the ICM cells on days 9 and 10, respectively).

### IL6 supplementation increases PE cell numbers in cultured blastocysts

**Fig. 3:**
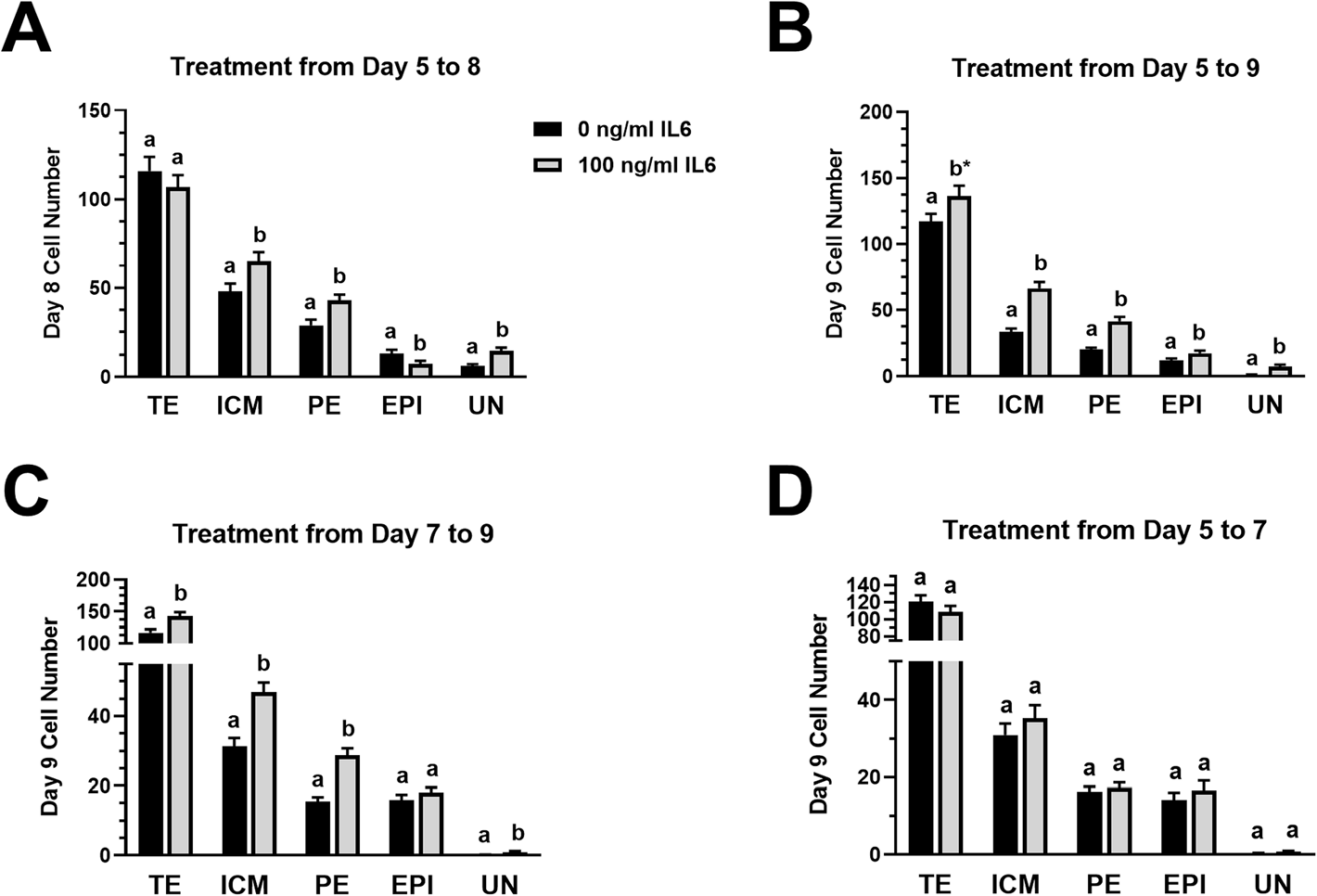
IL6 supplementation preferentially increases PE cell numbers. Blastocysts were cultured in SOF-BE1 supplemented with either 0 or 100 ng/ml IL6 for various periods of time, then immuno-staining for PE (GATA6^+^), EPI (NANOG^+^) and TE (CDX2^+^) was completed. All NANOG^+^/GATA6^+^ dual-positive cells were considered UN cells. **Panel a***:* IL6 supplementation from days 5 to 8 (*n* = 28–29 blastocysts/group over 3 replicate studies). **Panel b***:* IL6 supplementation from days 5 to 9 (*n* = 46–52 blastocysts over 4 replicate studies). **Panel c***:* IL6 supplementation from days 7 to 9 (*n* = 39–42 blastocysts/group over 3 replicate studies). **Panel d***:* IL6 supplementation from days 5 to 7 then removal of embryos from IL6 treatments and culture in the absence of IL6 from days 7 to 9 (*n* = 29–42 embryos/group over 2 replicate studies). Different superscripts for each cell type within each panel indicate differences (*P* < 0.05)

Several studies were completed to explore how IL6 supplementation influences EPI and PE development in bovine blastocysts (Fig. 3). Supplementing 100 ng/ml IL6 from day 5 to 8 produced blastocysts with greater (*P* < 0.05) total ICM cell numbers, but TE cell numbers were unchanged (*P* > 0.05) (Fig. 3a). The number of EPI cells was reduced (*P* < 0.05) and PE cell numbers were increased (*P* < 0.05) with IL6 supplementation. There also was an increase (*P* < 0.05) in UN cells following IL6 treatment. When examining ICM cells based solely on their marker expression, no changes in NANOG^+^ cell numbers (includes both EPI and UN cells) were detected with IL6 supplementation (22 ± 3.2 cells for IL6-treated vs 19.2 ± 2.5 cells for non-treated blastocysts) but an increase (*P* < 0.05) in GATA6^+^ cells (includes both PE and UN cells) were detected with IL6 supplementation (57.7 ± 4.1 cells for IL6-treated vs 35.0 ± 4.0 cells for non-treated blastocysts).

A separate study examined outcomes following exposure to 0 and 100 ng/ml IL6 from day 5 to 9 post-fertilization (Fig. 3b). We extended the culture period to day 9 because the previous study showed day 8 IL6-treated ICMs had a large portion of UN cells (~ 22% of ICM cells, see Fig. 3a). In day 9 blastocysts, IL6 supplementation increased (*P* < 0.05) the number of total ICM cells, EPI cells, PE cells and UN cells, and tended (*P* = 0.08) to increase TE cell numbers. Also, IL6 supplementation increased (*P* < 0.05) the number of NANOG^+^ cells (25 ± 2.1 cells for IL6-treated vs 13.3 ± 1.3 cells for non-treated blastocysts) and GATA6^+^ cells (48.8 ± 4 cells for IL6-treated vs. 21.4 ± 1.5 cells for non-treated blastocysts).

Additional studies were designed to further define the time point when IL6 influences total ICM cell numbers and ICM lineage distributions. In one study, IL6 was administered after blastocyst formation (between day 7 and 9) (Fig. 3c). Increases (*P* < 0.05) in ICM and TE cell numbers were detected following this 2-day IL6 supplementation. An increase (*P* < 0.05) in PE cell numbers was observed with IL6 supplementation whereas EPI numbers remained unchanged. An increase (*P* < 0.05) in UN cells was also detected in IL6-supplemented blastocysts, although the number of these cells were small in this study. In another study, IL6 was supplemented between day 5 and 7 post-fertilization, then embryos were removed from the IL6 treatment and maintained until day 9 in untreated medium (Fig. 3d). The number of ICM, TE, EPI, PE and UN cells at day 9 were not different from controls when embryos were removed from IL6 supplementation at day 7 (Fig. 3d). The IL6-dependent outcomes in this study were not different between embryos classified as morulae or blastocysts at day 7 (data not shown). However, regardless of IL6 treatment, day 9 blastocysts that were morulae on day 7 had fewer (*P* < 0.05) total ICM cells with fewer (*P* < 0.05) EPI and PE cells and greater (*P* < 0.05) numbers of UN cells when compared with embryos that were blastocysts at day 7 (data not shown).

### PE is the primary target for IL6-dependent STAT3 activation

**Fig. 4:**
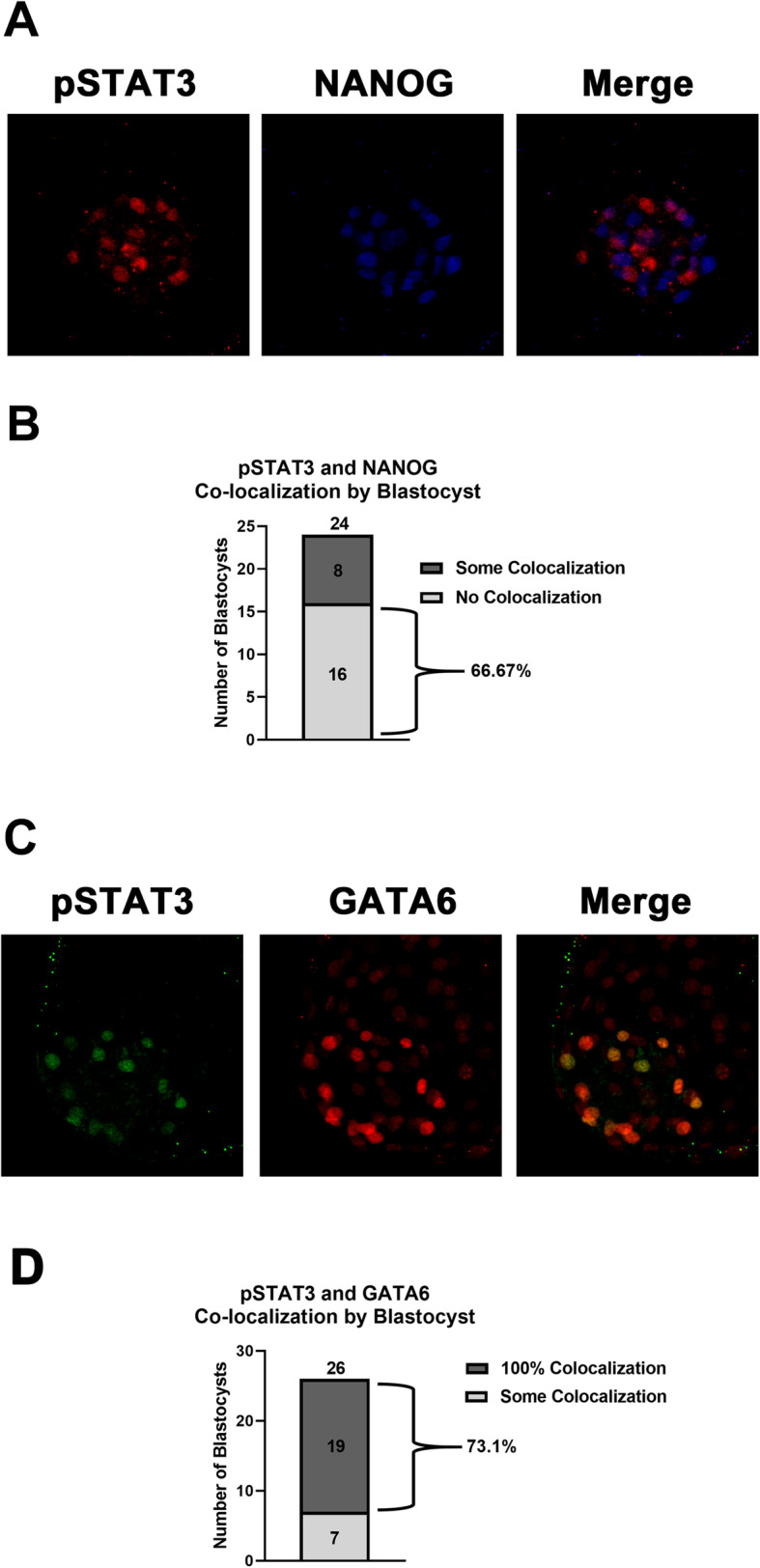
IL6 preferentially stimulates pSTAT3^Y705^ nuclear localization in PE cells. Day 9 blastocysts were treated with 100 ng/ml IL6 for 30 min before fixation and fluorescent immunostaining. **Panel a***:* Example of a blastocyst immunostained for pSTAT3^Y705^ (red) and NANOG (blue). Dual-positive nuclei will be purple. **Panel b***:* Number and percentage of blastocysts with either some or all ICM nuclei staining for both NANOG and pSTAT3^Y705^ (*n* = 24 pSTAT3^Y705^ positive blastocysts over 4 replicate studies) **Panel c***:* Example of a blastocyst immunostained for pSTAT3^Y705^ (green) and GATA6 (red). Dual-positive nuclei will be yellow. **Panel d***:* Number and percentage of blastocysts with either some or all ICM nuclei staining for both GATA6 and pSTAT3^Y705^ (*n* = 26 pSTAT3^Y705^ positive blastocysts over 3 replicate studies)

Phosphorylation and nuclear location of STAT3 is controlled by IL6 within the ICM of bovine blastocysts [3], but the specific cell lineage that responds to IL6 after EPI and PE specification has not been defined. Thus, the nuclear localization pattern of pSTAT3^Y705^ in day 9 IL6-treated blastocysts was examined (Fig. 4). Minor NANOG and pSTAT3^Y705^ co-nuclear localization was detected (Fig. 4a). Two-thirds of all blastocysts examined contained no detectable co-localization (Fig. 4b). Only 13.1 ± 5.6% of pSTAT3^Y705+^ cells were also stained positive for NANOG. By contrast, substantial pSTAT3^Y705^ co-nuclear localization occurred with GATA6 (Fig. 4c). In a majority of the blastocysts, every GATA6 positive ICM cell also was also positive for pSTAT3^Y705^ (Fig. 4d). Overall, 94.4 ± 1.7% GATA6 cells were positive for pSTAT3^Y705+^.

### Inhibiting JAK2 activity alters ICM cell numbers and cell lineage distributions

**Fig. 5:**
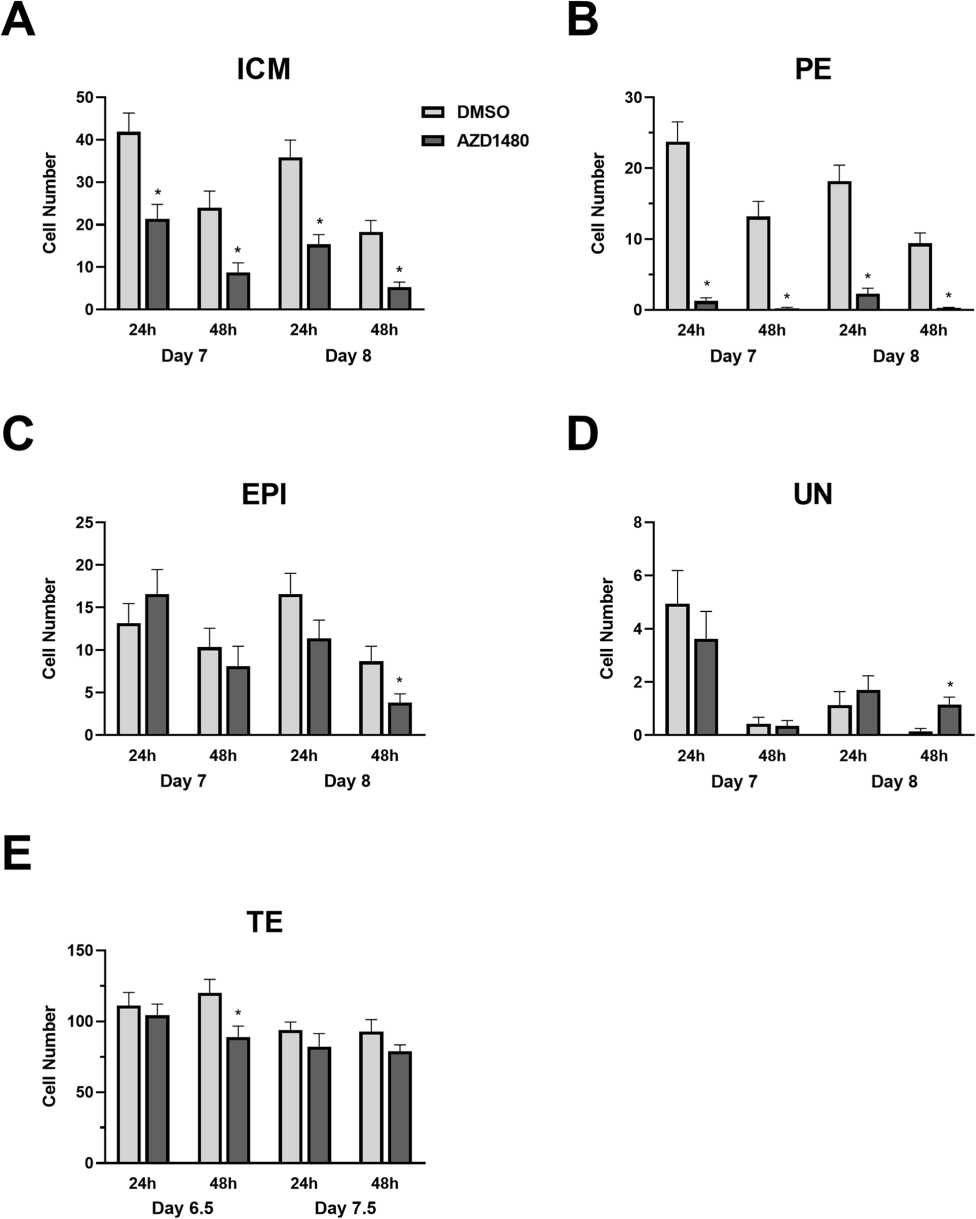
JAK2 inhibition reduces PE cell numbers. Either regular blastocysts collected on day 7 post-fertilization or expanded blastocysts collected on day 8 were exposed to 0 (DMSO only) or 3 μM AZD1480 for either 24 or 48 h then were fixed and immunostained for PE (GATA6^+^), EPI (NANOG^+^) and dual-positive UN cells (NANOG^+^/GATA6^+^) (*n* = 14–25 blastocysts/group over 3 replicate studies). The panels indicate total ICM (**Panel a**), PE (**Panel b**), EPI (**Panel c**), UN (**Panel d**), and TE cell numbers (**Panel e**) of day 7 and 8 blastocysts exposed to treatment for 24 or 48 h. Presence of an asterisk indicates a difference between AZD1480 treated and non-treated groups within each day and duration of treatment exposure (*P* < 0.05)

Pharmacological inhibition of JAK2 by AZD1480 effectively prevents STAT3 activity in bovine embryos [3, 13]. This inhibitor was used herein to examine how interruption of STAT3 and potentially other JAK2-mediated signaling molecules affects ICM cell numbers in bovine blastocysts (Fig. 5). The JAK inhibitor was provided for 24 or 48 h beginning either at day 7 or 8 post-fertilization. Representative images of blastocysts after exposure to these treatments are available as a supplement (Supplemental Figure 1). Fewer (*P* < 0.05) ICM cells were observed in JAK2 inhibitor-treated embryos at both days and exposure durations (Fig. 5a). The JAK2 inhibitor decreased (*P* < 0.05) PE cell numbers at both days and exposure durations (Fig. 5b) whereas TE, EPI and UN cell numbers were largely unaffected by exposure to this inhibitor (Fig. 5c-e).

## Discussion

Bovine ICM cells, like mouse and human ICM cells, have the plasticity to form either EPI or PE as they are beginning to undergo lineage determination, but they lose this plasticity once they have committed to one of the two cell fates [18, 19]. A MAPK effector kinase (MEK)-dependent signaling event controls this cell fate decision. Embryokines that activate MEK (e.g. FGF2/4) will not greatly influence total ICM cell numbers but will cause more ICM cells to commit to the PE lineage than the EPI lineage. The opposite is true with MEK signal interference, where more ICM cells will commit to the EPI lineage than the PE lineage. Other factors involved in ICM lineage development in the bovine are largely unknown, although recent work by this laboratory showed that IL6 increases ICM cell numbers in bovine blastocysts [3, 5]. This led us to speculate that IL6 may also be involved with EPI and/or PE lineage development.

This work determined that IL6 supplementation increases PE cell numbers in bovine blastocysts. A reduction in PE cell numbers was observed in blastocysts over time in culture, but IL6 supplementation was able to cause an approximate doubling in PE cells regardless of the day of culture. Conversely, the responses of EPI cells to IL6 were not consistent across studies. In one study, IL6 increased EPI cell number, another detected a decrease, and a third found no effect. The reason for these differences may lie in the different IL6 supplementation scheme used (starting at day 5 or 7) or the blastocyst age when sampled (day 8 or 9). When IL6 supplementation began on day 5, a decrease in EPI cell number was observed in day 8 blastocysts, while an increase was observed on day 9. Intriguingly though, in the day 8 blastocysts, no change was observed in the number of NANOG^+^ cells with IL6 treatment, indicating a lot of ICM cells were still NANOG and GATA6 dual-positive (UN). This may indicate that early treatment of IL6 (prior to blastocyst formation) slows EPI differentiation, before stimulating a small but significant increase. Indeed, IL6 had no effect on EPI cell number when supplementation began after blastocysts had already formed (day 7). Alternatively, these discrepant outcomes on EPI cell number may simply be a result of chance. IL6 effects on the PE were dramatic and consistent, while its effects on the EPI were small. This is an important consideration because it infers that IL6 is not functioning to control EPI and PE fate determination. If it were, an increase in the population of one cell type would be evident at the expense of the other, similar to what is seen with FGF/MEK manipulation work [18, 19]. Instead, we propose that IL6 is mediating PE development after its specification. Work was not completed to understand if IL6 functions by promoting PE cell proliferation or inhibiting PE cell death.

This work also showcases how rapidly ICM cells respond to exogenous IL6. Increases in ICM and PE cell numbers were observed after just 24 h of supplementation, and STAT3 activation and nuclear localization could be observed in just 30 min. The rapidity of IL6 responses likely signifies that IL6 itself is acting upon the ICM cells, although one question that remains unanswered is how IL6 traverses the TE layer to act on the ICM. The TE contains a tight junction barrier that restricts the movement of molecules. Transcytosis is a possible mechanism for IL6 transport given that the TE contains both subunits of the IL6 receptor (*IL6R* and *IL6ST*) [20]. Alternatively, there is evidence that IL6 can modify tight junction permeability to permit its passage through the intestinal epithelium [21, 22], and perhaps a similar mechanism is utilized in the embryo. However, it remains possible that IL6 does not pass through the TE but instead acts on the TE to stimulate an effector molecule that acts on the ICM. Further work is needed to describe the exact mechanism responsible for this IL6 effect on the ICM.

It was interesting to observe that IL6 supplementation caused an increase in UN cell numbers at day 9. This could reflect IL6’s ability to improve ICM cell numbers prior to EPI/PE specification, as observed recently in work completed on blastocysts examined at day 7 post-fertilization [3]. Alternatively, it may suggest that IL6 can limit or delay cell differentiation. There also was a decrease in UN cell numbers, regardless of IL6 supplementation, after day 8. These UN cells are thought to differentiate into EPI or PE cells by day 9 of bovine blastocyst development [19]. However, no improvements in EPI or PE numbers were detected at days 9 and 10 when compared with numbers at day 8. Thus, UN cell differentiation may have only partially replaced EPI and/or PE cells that had been lost. Alternatively, UN cell apoptosis may have occurred. This is a normal occurrence for UN cells that persist within the ICM [23].

It also was interesting that none of the observed effects on total ICM, PE, EPI and UN cell numbers were detected in embryos that were exposed to IL6 from day 5 to 7 and thereafter were cultured without IL6 until day 9. This indicates that supplemental IL6 must be present on days 8 and 9 to observe effects on PE and UN cells.

Another important finding of this work established a role for JAK2 in mediating PE cell abundance within the bovine ICM. Earlier work established that pSTAT3 activation could be detected in nearly every ICM cell within the blastocyst at day 8 [3]. This work observed that by day 9 pSTAT3 activation co-localized with PE cells more so than EPI cells. Moreover, chemical inhibition of JAK2 activity, which prevents pSTAT3 activation, does not largely influence EPI and UN cell numbers but profoundly reduces PE cell numbers. Also, this inhibition occurred regardless of whether the inhibitor is administered before or during EPI/PE lineage specification (days 7 and 8, respectively) and regardless of whether embryos are exposed for 24 or 48 h. Lower concentrations of AZD1480 were used for this work than were used in previous work (3 vs 5 μM) because work in other tissues suggested that AZD1480 could inhibit aurora kinases when provided at ≥5 μM [24].

Collectively, this work indicates that JAK2-dependent signals control PE development in cattle. This finding is similar to what has been observed in the mouse. JAK-dependent events control PE cell development in the mouse blastocyst and in PE cells developed from primed murine embryonic stem cells (ESCs) [25, 26]. This work also observed a strong co-expression of GATA6 and pSTAT3 and a weak co-expression of NANOG and pSTAT3 in murine blastocysts. Also, JAK inhibition prevented PE development in murine ESCs, and the absence of this signal increases the rate of PE apoptosis. Therefore, all indications are that JAK-dependent signals are needed for PE development in both the bovine and mouse blastocyst. STAT3 is the likely JAK-dependent signal controlling IL6 effects within the ICM.

Although considerable overlap in the control of PE lineage development exists in the mouse and cow, the two species utilize different IL6 cytokine family members to control PE development. LIF is the primary embryokine controlling mouse PE development. LIF acts through JAK-dependent signals to increase murine PE cell numbers within blastocysts and in PE cell lines derived from primed mouse ESCs [25, 26]. Moreover, LIF acts primarily by limiting PE cell apoptosis [25, 26]. Lastly, LIF does not affect EPI cell numbers, indicating that LIF likely functions primarily to promote the expansion of PE cells and not EPI/PE cell fate determination. By contrast, there is no evidence suggesting that LIF can affect PE cell numbers in bovine blastocysts. The ligand-specific subunit for LIF (termed LIFR) is lowly expressed in bovine blastocysts [3]. Also, LIF supplementation does not affect ICM cell numbers in bovine blastocysts [27]. In fact, in one study, exposure to LIF reduced the incidence of hypoblast formation in bovine blastocysts placed into extended cultures [27]. Thus, it appears that the common IL6ST receptor subunit is being utilized to control JAK2-dependent signals within the PE in both the bovine and mouse blastocyst, but the specific IL6 family member that controls this activity differs between the two species.

## Conclusions

In conclusion, this work provides compelling evidence that IL6 manipulates ICM development after EPI/PE cell fates are established. The PE cells are the target for IL6, where a JAK-dependent signal is used to increase PE numbers. This JAK2-dependent signal is required for PE development after the PE lineage has been specified in the bovine blastocyst. The necessity of IL6 for PE development while the bovine embryo is developing within the uterus remains unknown, but a subset of IVP bovine embryos experience defective yolk sac development after their transfer [28, 29], and perhaps insufficient IL6 signaling during IVP embryo production compromises PE development in ways that cause some pregnancies to fail after IVP embryos are transferred.

## Methods

No animals were used for this work. All studies were completed on slaughterhouse-derived materials from a commercial slaughterhouse that followed humane slaughter practices according to USDA guidelines. Reagents were purchased from ThermoFisher Chemical Company (Waltham, MA), unless otherwise specified.

### In vitro embryo production

Bovine blastocysts were produced by in vitro maturation, fertilization and culture procedures described previously [5, 30]. In brief, cumulus-oocytes complexes (COCs) were extracted from slaughterhouse-derived ovaries (Brown Packing, Gaffney, SC). For fertilization, COCs were exposed to live spermatozoa (1 × 10^6^ spermatozoa/ml medium) from pooled semen from four Holstein bulls (donation from Select Sires, Plain City, OH, USA) using a biphasic gradient (40 and 80% [v/v] Bovipure™; Nidacon; Spectrum Technologies, Healdsburg, CA, USA). After 14–18 h at 5% CO_2_ in air at 38.5 °C, presumptive zygotes were denuded by gentle pipetting and placed in groups of ~ 25 in 50 μl SOF-BEI drops under light mineral oil and incubated in 5% CO_2_, 5% O_2_ and 90% N_2_ in humidified air at 38.5 °C [31]. The day of fertilization was designated as day 0. The embryos were cultured in SOF-BEI until day 7, 8, 9, or 10, as specified for each experiment.

### IL6 supplementation

In most studies, treatments were administered to existing drops by addition of 2 μl of concentrated recombinant bovine IL6 (KingFisher Biotech, St. Paul, MN, USA) prepared in SOF-BEI medium. The control treatment was composed of carrier only (1% [w/v] bovine serum albumin [BSA]). Embryos were then maintained in their original drops until harvested for analysis. In one study, day 7 blastocysts were collected from their existing drops, and individual blastocysts were placed into 50 μl SOF-BEI containing 0 or 100 ng/ml IL6. In another study, embryos that had been exposed to IL6 treatments beginning on day 5 were removed from their drops on day 7, washed twice in SOF-BEI and placed into non-IL6-treated drops (50 μl SOF-BEI; 2–7 embryos/drop).

### JAK2 inhibition

A stock solution of 100 mM AZD1480 (JAK 2 inhibitor; S2162; Selleck Chemicals, Houston, TX, USA) was prepared with DMSO as the carrier (stored at − 80 °C). Blastocysts were removed from their original drops on day 7 or 8 post-fertilization and placed into 50 μl SOF-BE1 containing either 3 μM AZD1480 or carrier only (0.003% DMSO). Blastocysts were collected and processed for immunofluorescence after 24 or 48 h.

### Immunofluorescence

Procedures were completed as described previously, with some modifications [3, 5]. Differential staining for TE and ICM cells in blastocysts was completed as described previously [5] using mouse anti-Caudal Type homeobox 2 (CDX2; Biogenex, San Ramon, CA, sold ready-to-use), anti-mouse IgG (Alexafluor 488; 1:200), and 4′,6-diamidino-2-phenylindole (DAPI; 1 μg/ml).

For CDX2, NANOG and GATA6 co-staining studies, blastocysts were permeabilized with 0.5% Triton-X in Dulbecco’s Phosphate Buffered Saline (DPBS) for 30 min, then blocked with 10% [v/v] horse serum for 1 h. Due to antibody overlap, two rounds of primary and secondary antibody incubations were completed. First, blastocysts were incubated with rabbit anti-GATA6 (Cell Signaling Technology, Danvers, MA; 5851 T; 1:500) and mouse anti-NANOG (eBioscience; 14–5768-82; 1:200) for 1 h at room temperature, then blastocysts were incubated with donkey anti-rabbit IgG (Alexafluor 555; 1:200) and donkey anti-mouse IgG (Alexafluor 647; 1:200). Blastocysts were then incubated with mouse anti-CDX2 antibody (same as above) for 1 h at room temperature and finally exposed to donkey anti-mouse IgG (Alexafluor 488; 1:500).

For pSTAT3^Y705^ co-staining with NANOG or GATA6, blastocysts were incubated with 70% [v/v] ethanol for 5 min at room temperature then were blocked with 10% horse serum containing 0.5% Triton-X for 1 h at room temperature. Blastocysts were then incubated for 1 h at room temperature or overnight at 4 °C with either rabbit anti-pSTAT3^Y705^ (Cell Signaling Technologies; 9145 T; 1:100) and mouse anti-NANOG (same as above; 1:200), or with mouse anti-pSTAT3^Y705^ (Santa Cruz Biotechnology, Dallas, TX; sc-8059; 1:200) and rabbit anti-GATA6 (same as above; 1:500). After washing, blastocysts were incubated with donkey anti-mouse IgG (Alexafluor 488 or 647; 1:200) and anti-rabbit IgG (Alexafluor 555; 1:200).

After each of these staining procedures, embryos were incubated with 1 μg/ml DAPI for 5 min at room temperature then washed in PBS-PVP and flattened on a glass slide lined with petroleum jelly. Staining was visualized with an Eclipse Ti-E inverted microscope equipped with an X-cite 120 epifluorescence illumination system and DS-L3 digital camera. Images were captured with NIS-Elements Software (Nikon Instruments, Melville, NY), and cell counting was completed with the cell counter plugin in the program FIJI (ImageJ) [5]. Some embryos presented in figures may appear larger than others. This is a result of variable pressure applied to each coverslip as embryos were “flattened”. All embryos were imaged at 20X magnification. A representative sampling of regular, expanded and hatched blastocysts was selected on day 8. At day 9, only expanded hatched blastocysts were selected for analysis, and at day 10, only hatched blastocysts were selected. In some studies where blastocysts were only stained with anti-CDX2 antibody and DAPI, CDX2^+^:DAPI^+^ nuclei were considered TE, while CDX2^−^:DAPI^+^ nuclei were considered ICM cells. This method and anti-CDX2 antibody are commonly used to determine ICM and TE cell numbers in bovine blastocysts [32–35].

### Statistical analyses

All analyses were completed using the Statistical Analysis System (SAS for Windows; SAS Institute Inc., Cary, NC, USA). Three to four replicates were completed for most studies. For all analyses, embryo was considered the experimental unit. Replicate was considered a random, independent variable. Differences in cell number were analyzed by least-squares ANOVA, using the general linear model (Proc GLM). In one study, ICM cell number data were cube-root transformed before analysis as they contained a right-tail distribution. Individual comparisons were partitioned further by using the Probability of Difference (PDIFF) test in SAS. Statistical significance was determined at *P* ≤ 0.05.

## Supplementary Information

**Additional file 1: Supplementary Figure 1**. Example images of blastocysts treated with either 0 (DMSO) or 3 μM AZD1480. Day 8 blastocysts were exposed to control (DMSO) or AZD1480 treatments, then were fixed and immunostained using markers for TE (CDX2; green nuclei), PE (GATA6; red nuclei), and EPI (NANOG; blue nuclei).

## Data Availability

All data supporting the results reported in this article can be found within the article. All data will be made available upon request.

## Abbreviations

CDX2: Caudal type homeobox 2
ICM: Inner cell mass
EPI: Epiblast
FGF: Fibroblast growth factor
FGFR: Fibroblast growth factor receptor
GATA6: GATA binding protein 6
IL6: Interleukin-6
IL6R: Interleukin-6 receptor
IL6ST: Interleukin-6 signal transducer (aka gp130)
IVP: In-vitro produced
JAK: Janus kinase
LIF: Leukemia inhibitory factor
LIFR: Leukemia inhibitory factor receptor
MAPK: Mitogen-activated protein kinase
PE: Primitive endoderm
PI3K: Phosphoinositide 3-kinase
SOF: Synthetic oviduct fluid
STAT3: Signal transducer and activator of transcription 3
TE: Trophectoderm
UN: Undifferentiated ICM
