# Supplementary figures and images for: Interleukin-6 promotes primitive endoderm development in bovine blastocysts
Source: BMC Dev Biol. 2021 Jan 12;21:3. doi: 10.1186/s12861-020-00235-z (PMC7802221; doi:10.1186/s12861-020-00235-z)

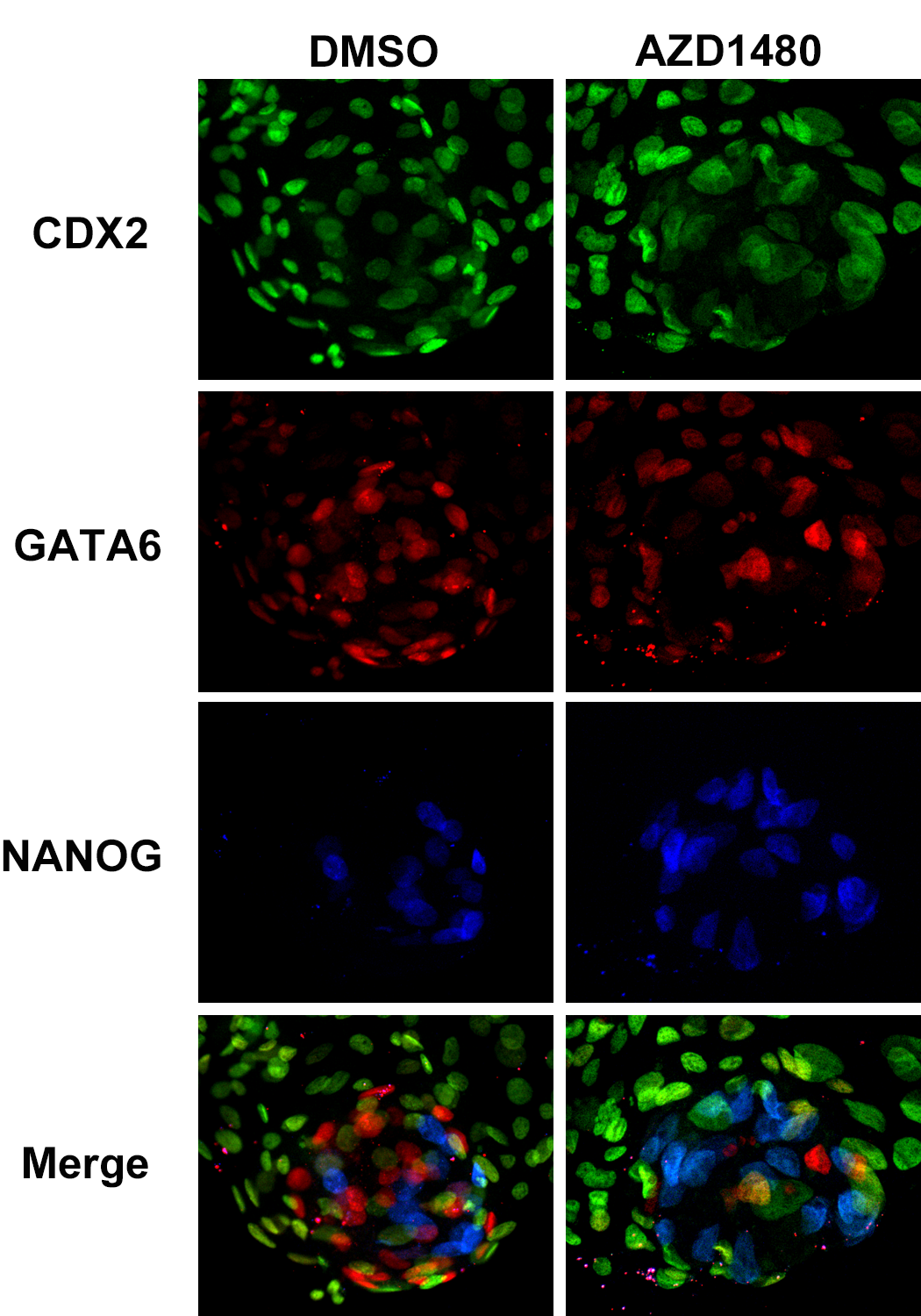

Supplement: Supplementary file 1 — Additional file 1: Supplementary Figure 1. Example images of blastocysts treated with either 0 (DMSO) or 3 μM AZD1480. Day 8 blastocysts were exposed to control (DMSO) or AZD1480 treatments, then were fixed and immunostained using markers for TE (CDX2; green nuclei), PE (GATA6; red nuclei), and EPI (NANOG; blue nuclei). [file 12861_2020_235_MOESM1_ESM.tiff]
